# Chemistry, Technology and Utilization of Nanolime

**DOI:** 10.3390/ma18214846

**Published:** 2025-10-23

**Authors:** Yaroslav Yakymechko, Roman Jaskulski, Daria Jóźwiak-Niedźwiedzka, Maciej Banach

**Affiliations:** 1 Institute of Chemistry and Chemical Technologies, Lviv Polytechnic National University, 13 Bandera St., 79000 Lviv, Ukraine; yayakym@gmail.com; 2 Department of Civil Engineering, Wrocław University of Environmental and Life Sciences, 24 Grunwaldzki Sq., 50-363 Wrocław, Poland; 3 Institute of Fundamental Technological Research, Polish Academy of Sciences, Pawińskiego 5b, 02-106 Warsaw, Poland; djozwiak@ippt.pan.pl; 4 Institute of Civil Engineering, Warsaw University of Technology, 17 Łukasiewicza St., 09-400 Płock, Poland; maciej.banach@pw.edu.pl

**Keywords:** nanolime, nanolime synthesis, cement-based materials, CO_2_ capture, self-healing, heritage conservation

## Abstract

This article provides a comprehensive review of the chemistry, production technology, and utilization of nanolime. Particular attention is given to the synthesis of Ca(OH)_2_ nanoparticles through both bottom-up and top-down approaches, highlighting how modern techniques enable precise control of particle size, morphology, and stability. The physicochemical properties of nanolime are discussed in relation to its role as a highly reactive, multifunctional additive, i.a., for cementitious systems, asphalt, and autoclaved products. Its applications are explored with emphasis on performance improvement in construction engineering, including enhanced strength, durability, self-healing potential, and compatibility with low-carbon binders. Beyond construction, nanolime is also examined as a material with relevance to environmental protection, CO_2_ sequestration, and heritage conservation. The review demonstrates that nanolime is a versatile and strategic material whose properties can be tailored to specific engineering needs, though challenges such as agglomeration, carbonation control, scalability, and long-term durability remain. Future research directions are outlined, focusing on sustainable production methods, functional integration into next-generation binders, and cross-disciplinary applications.

## 1. Introduction

Lime is one of the oldest known construction materials, with evidence of its use dating back at least ten millennia. For millennia, calcium oxide and its hydrated form, calcium hydroxide, have been indispensable binders for mortars, plasters, and other applications in architectures across the world [1]. Construction lime, along with clay and gypsum, is one of the oldest binders used from prehistoric times to the present day. In the modern era, lime remains relevant, not only for restoration and conservation but also as a component of sustainable construction technologies.

**Figure 1:**
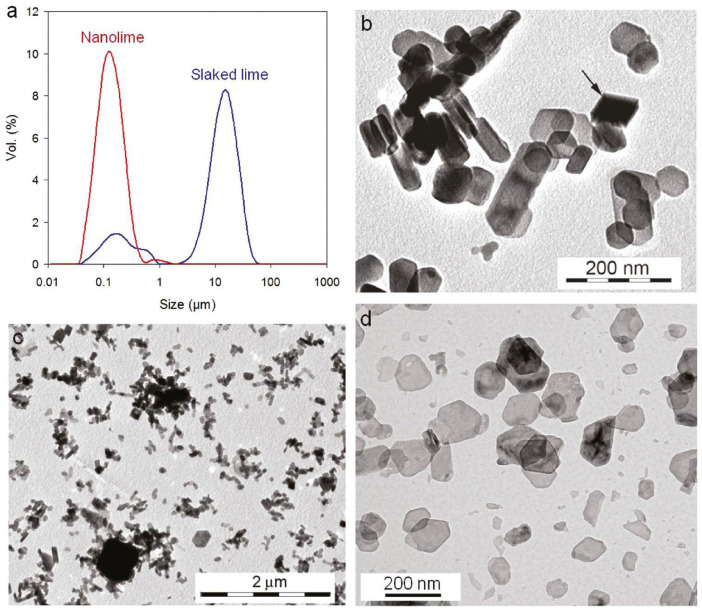
Particle size distribution and morphology of nanolime vs. conventional slaked lime. (**a**) Nanolime sols contain Ca(OH)_2_ crystals in the tens of nanometers (red peak), whereas slaked lime suspensions are dominated by micron-scale particles (blue peak). TEM micrographs show (**b**,**c**) the heterogeneous, aggregated plate-like particles in traditional slaked lime, versus (**d**) the uniform, well-dispersed nanolime particles [2] (licenced under CC BY-NC-ND 4.0).

The emergence of nanotechnology has revitalized interest in lime by enabling the production of nanolime, defined as dispersions of calcium hydroxide particles typically 50–300 nm in size [2,3,4] (Figure 1). The nanoscale dimension imparts unique physicochemical properties: a large specific surface area, enhanced reactivity, and improved penetration into porous matrices compared to traditional lime [5,6]. These attributes make nanolime especially promising for construction-related applications where durability, compatibility, and environmental performance are critical [2,6].

The great interest in nanolime is caused by the fact that Ca(OH)_2_ nanoparticles have some properties that are not found in conventional industrial lime. In particular, when nanolime is applied to various surfaces in a thin layer, it undergoes very rapid carbonisation and forms strong and hard layers. Although the rate and extent of carbonation depend strongly on relative humidity and CO_2_ concentration, as demonstrated in several studies [7,8], this property of nanolime is used for the restoration and refurbishment of ancient monuments, buildings and wall paintings that were built using natural limestone or marble [5,9,10]. Another massive use of nanolime is as a chemical adsorbent, for absorbing carbon dioxide and other harmful gases, as well as for cleaning and disinfecting wastewater [11,12]. Of great interest are the studies that provide data on the use of nanolime as a material for energy storage [13,14,15,16,17,18,19,20]. We should also mention the use of calcium nanohydroxide in medicine as an antibacterial substance and a component that regulates the acid balance in the gastrointestinal tract. Calcium hydroxide is a drug that is often used for endodontic surgery and other dental procedures [21,22,23].

However, in cement-based materials, nanolime serves multiple functions. As a nano-additive, it accelerates cement hydration, refines microstructure, and improves early-age strength [24,25,26]. Its high surface energy provides abundant nucleation sites for calcium silicate hydrate (C–S–H) formation, enhancing strength development and reducing porosity. In blended and low-clinker binders [27], nanolime compensates for reduced calcium hydroxide availability, improving pozzolanic reactions with supplementary cementitious materials such as fly ash, slag, or calcined clays [26]. This dual role as a reactivity enhancer and durability promoter, positions nanolime as a key material for low-carbon cement technologies.

Nanolime has also been studied in lime–pozzolan and lime–silica systems, where its fine particle size improves reaction kinetics, leading to denser and more durable matrices [27]. In autoclaved aerated concrete, it can act as a reactive lime source that accelerates tobermorite formation, enhancing mechanical performance and dimensional stability [28].

In the asphalt industry, nano-Ca(OH)_2_ has been shown to enhance resistance to moisture-induced damage, rutting, and fatigue cracking [29,30]. By improving adhesion between aggregate and bitumen, nanolime reduces stripping and extends pavement life, offering a sustainable alternative to conventional anti-stripping agents [31,32]. Its role as a filler also contributes to improved stiffness and long-term mechanical performance of asphalt mixtures [33,34,35,36].

Another emerging frontier is self-healing construction materials. Due to its ability to rapidly carbonate, under suitable environmental conditions, nanolime can carbonate and precipitate CaCO_3_ within cracks, sealing microfractures and improving durability when relatively concentrated nanolime dispersions are applied [37,38]. Several recent studies have explored self-healing mechanisms in lime- and nanolime-based systems. For instance, in mortars incorporating vascular networks filled with nanolime, dissolved calcium species can re-crystallize and seal microcracks [39]. Likewise, historic lime mortars have demonstrated self-healing capacity through the dissolution and re-precipitation of calcium compounds under moist conditions [40].

When embedded in cementitious matrices, nanolime nanoparticles can serve as internal “healing agents,” promoting localized mineralization [39]. This concept aligns with the broader push toward autonomous, low-maintenance building materials with extended service lives.

Despite these advances, several challenges limit widespread adoption of nanolime in construction. Particle agglomeration [41], uneven distribution [42], and incomplete carbonation under unfavorable curing conditions can compromise performance [43,44]. Long-term durability studies in aggressive environments (e.g., freeze–thaw cycles, sulfate exposure) are still limited [9,45], and the economics of large-scale nanolime production remain an obstacle compared to conventional additives [2]. Nevertheless, ongoing progress in synthesis methods (both chemical precipitation and mechanical activation) and the growing availability of commercial products suggest that nanolime could soon transition from niche applications to broader use in the construction sector [46].

Overall, nanolime exemplifies how traditional materials can be reengineered through nanotechnology to meet the demands of both heritage preservation and modern sustainable construction. Its unique chemistry and versatility, spanning cement-based materials, asphalt, and self-healing systems, highlight its potential as a transformative construction material.

This review synthesizes current knowledge on the chemistry, technology, and utilization of nanolime, emphasizing its applications in construction engineering and identifying pathways for future research and practical implementation.

## 2. Methods of Nanolime Synthesis

### 2.1. Overview and Classification of Synthesis Routes

To understand the unique performance of nanolime in various applications, it is first essential to examine how it is produced. This section outlines the main synthesis methods, their underlying mechanisms, and the technological factors that determine particle size, purity, and stability.

The technology of nanolime production has evolved considerably in the past two decades, driven by the need to obtain well-dispersed, reactive Ca(OH)_2_ nanoparticles suitable for construction and conservation applications. Several synthesis routes have been developed, broadly classified into bottom-up chemical precipitation methods and top-down mechanical activation methods [47,48,49], with additional hybrid and bio-inspired approaches emerging.

Bottom-up methods involve the formation of Ca(OH)_2_ nanoparticles from molecular or ionic precursors. This process mostly occurs through self-assembly or catalytic chemical reactions. In nanolime production technologies, this method is carried out through various chemical reactions that produce highly dispersed calcium hydroxide and other by-products [50,51,52], or by hydrating CaO in a special environment [4] or by adding certain lime quenching catalysts [53].

Top-down nanotechnology is a technology for producing nanostructured materials in which nanometric particle size is achieved by grinding large particles, powders or grains of a solid. This method starts with bulk large-size particles, which are subsequently transformed into nanostructures by physical, chemical and mechanical processes. This method is realised by grinding in ball planetary mills [54] or by using highly efficient vibration processing methods [55]. Other top-down approaches include laser ablation and thermal decomposition, mechanical homogenisation by stirring and ultrasonication, as well as hydrogen plasma–metal reactions, which are also used for the preparation of nanostructured calcium hydroxide materials [56,57,58,59].

### 2.2. The Synthesis Routes and Technological Aspects of Nanolime Production

#### 2.2.1. Bottom-Up Methods

Zhernovsky et al. [50] studied phase formation products in a gypsum–lime binder system. In the CaO-SO_3_-CO_2_-H_2_O system, each component—lime and semi-aqueous gypsum (basanite)—harden according to individual patterns without forming any ternary compounds. It is noted that, on the basis of a full-profile quantitative X-ray phase analysis, in addition to lime nanoparticles, microcrystals of the mineral rapidcreekite were found. The anisotropic profile of rapidcreekite exhibit a rather small crystallite size in the (001) direction. This allows us to consider the mineral formations as 1D nanoparticles with some palliation.

Samanta et al. [51] investigated a method for producing calcium hydroxide nanoparticles in a liquid medium at room temperature. This paper describes a simple and inexpensive method for the preparation of nanolime. The nanolime was obtained by the interaction of calcium nitrate dihydrate [Ca(NO_3_)_2_∙2H_2_O] and sodium hydroxide [NaOH]. A set of physicochemical analyses confirmed the formation of hydrated lime nanoparticles with a particle size of about 350 nm. The main advantage of this method is the relatively low energy consumption. The XRD and SAED results showed that the most intensive growth of Ca(OH)_2_ crystallites occurs along the (001) plane, as it is the most energetically favourable, with the formation of hexagonal calcium hydroxide plates.

Madrid & Lanzón [52] synthesised nanolime using the reaction:

2NaOH + CaCl_2_ → Ca(OH)_2_↓ + 2NaCl (1)

Their paper presents a fairly complete procedure for the preparation of Ca(OH)_2_ nanoparticles. The synthesis was carried out under a controlled atmosphere to avoid carbonation of the hydroxide by carbon dioxide in the air. In addition, a method for purifying the finished product from sodium chloride was developed. Figure 1 shows the scheme of such purification. It is shown that rapid analytical methods (pH, conductivity, chromatography) are suitable for assessing the purity of the finished product from NaCl.

The yield of high-purity Ca(OH)_2_ nanoproduct is 70% and it is very easily dispersed in 2-propanol. At the same time, water negatively affects the stability of nanoparticles due to the formation of Ca(OH)_2_ agglomerates. Agglomeration is caused by molecular interactions with the formation of hydrogen bonds between H_2_O and Ca(OH)_2_.

Daniele and Taglieri [4] obtained calcium hydroxide nanoparticles in the same process by precipitation from a sodium hydroxide and calcium chloride solution. To improve the properties of nanometric calcium hydroxide, a surfactant (Triton X-100) was added in certain proportions. The resulting nanoparticles have the regular shape of hexagonal plates with a diameter of less than 200 nm. It was found that with an increase in the amount of surfactant to the optimum, the particle size decreases to 20 nm.

The possibility of synthesising nanoparticles at ambient temperature by increasing the solubility of calcium during the formation of complexes with sucrose was studied by Martínez-Ramírez et al. [53]. The data of physicochemical analysis confirmed the formation of calcium hydroxide nanoparticles. The percentage of product yield was determined by thermogravimetric analysis. The optimal synthesis conditions were found at a temperature of 25 °C in a 5% sugar solution and a reaction time of 4 h. The yield of hexagonal calcium hydroxide particles with sizes ranging from 25 to 200 nm was 77%. The formation of portlandite is associated with the formation of complex compounds of calcium with sucrose, which increase the solubility of Ca(OH)_2_ several times.

Studies by Rodriguez-Navarro et al. [54,60] have shown that the microstructural particles of Ca(OH)_2_ in aged plaster coatings consist of lamellar primary nanoparticles that tend to aggregate into non-oriented clusters ranging in size from 100 nm to 1 micrometre. They are very similar to those found in commercial nanofuels. The authors of the study [54] synthesised nanolime in alcohol solutions (ethanol and 2-propanol). It was found that ethanol and 2-propanol react with calcium hydroxide to form calcium alkoxides (calcium ethoxide or calcium isopropoxide). The authors in paper [60] claim that the replacement occurs through the joint dissolution of calcium hydroxide and calcium alkoxides, with the original portlandite and the derived calcium alkoxides having the same properties. However, calcium alkoxides are unstable and gradually transform back into Ca(OH)_2_ over time. Their temporary presence can also affect the nucleation process of nanolime particles and, consequently, the kinetics of carbonation.

Zhang et al. [61] described an optimised process of Ca(OH)_2_ nanoparticle synthesis using an impinging stream co-precipitation method. They investigated the influence of selected process variables (i.a. dispersant type and dosage, circulating flow rate, reactant concentration ratio, reaction temperature) on the particle size. The results showed that the concentration ratio of reactants has a significant effect on the size of Ca(OH)_2_ nanoparticles and the average particle size prepared under optimal conditions was 107.67 nm. Microstructure analysis showed that the prepared Ca(OH)_2_ nanoparticles have high purity and a good crystal structure.

In order to obtain a highly permeable nanosuspension, calcium hydroxide nanoparticles were synthesized by the solvothermal reaction of metal calcium in water [62]. Metallic granular calcium and distilled water were used for the synthesis of nanoparticles. The synthesis was carried out in a reactor at a temperature of 90 °C. The nanolime particles were dispersed in ethanol, isopropanol, butanol, and water in different proportions. The results show that after an appropriate sonication of the suspension, colloidal dispersions in organic solvents are stable for up to 96 h. The size of the nanoparticles ranges from 40 to 800 nm.

**Figure 2:**
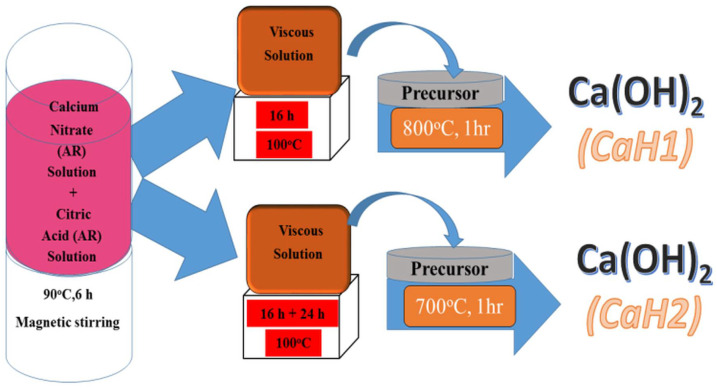
Scheme of the synthesis process of calcium hydroxide nanoparticles [63] (licenced under CC BY 4.0).

Singh et al. [63] used sol–gel technology to synthesize Ca(OH)_2_ nanoparticles. The synthesized nanoparticles have a tetragonal phase with a P3m1 space group. These nanoparticles are spherical in shape with sizes ranging from 55 to 69 nm. The nanolime was prepared as follows: a solution of calcium nitrate tetrahydrate was mixed in a stoichiometric proportion with a solution of citric acid, maintaining a ratio of cations to citric acid of 1:3. The resulting mixture was stirred in a magnetic stirrer for six hours. Nanoparticles of different sizes (CaH1 and CaH2) were obtained by annealing the solution at 800 °C and 700 °C, respectively. The scheme of the synthesis process is shown in Figure 2.

The schematic diagram in Figure 2 illustrates the main synthesis routes of nanolime, highlighting the differences between bottom-up and top-down approaches. As shown, precipitation methods enable fine control over particle size and purity, whereas mechanical activation provides scalability. This visual comparison emphasizes how synthesis route selection directly influences the resulting nanolime properties.

An efficient method for the synthesis of nanosized Ca(OH)_2_ (~90 nm) at room temperature for the mass production of nanodispersed lime was developed and presented in [64]. This was achieved by controlling the growth of Ca(OH)_2_ crystals in the $\left(\bar{1}010\right)$ (vertical) and $\left(0001\right)$ (horizontal) directions by adding octylphenylpolyoxyethylene ether (OPPE) and dimethylformamide (DMF) during the lime quenching reaction. Density functional theory (DFT) calculations have shown that the effect on the growth of Ca(OH)_2_ crystals is caused by the addition of OPPE to the $\left(0001\right)$ face and DMF to the $\left(\bar{1}010\right)$ face of Ca(OH)_2_. The experimental results showed that the synthesised nano-Ca(OH)_2_ has higher stability and carbonized faster than commercial Ca(OH)_2_.

Giuliana Taglieri et al. [65,66,67,68] have developed a new and original method for the preparation of Ca(OH)_2_ nanoparticles using an anion exchange resin. The method consists of mixing a 0.1 M aqueous solution of calcium chloride (CaCl_2_) with a calculated amount of anion exchange resin. The resulting mixture was kept at room temperature with constant stirring. The resulting product was separated from the resin particles by simple sieving. Ca(OH)_2_ nanoparticles were formed by the following reaction:

2(R-OH) + CaCl_2_→ 2(R-Cl) + Ca(OH)_2_ (2)

At the same time, calcium hydroxide was deposited on the surface of the resin particles, and diffused into the solution when stirred. According to TEM (Philips CM100), the particle size was less than 100 nm.

#### 2.2.2. Top-Down Methods

In addition to chemical methods for the production of Ca(OH)_2_ nanoparticles, i.e., the ‘bottom-up’ methods, it is proposed to use ‘top-down’ technology [55,69]. Eswaramoorthi et al. [69] used the method of grinding of a suspension of commercial lime in a planetary ball mill to obtain lime nanoparticles. The grinding time was about 12 h. Lime particles with sizes ranging from 100 to 350 nm were obtained.

Yakymechko et al. [55] developed a technology for vibration treatment of lime dough in a special vibrating apparatus with an oscillation frequency of 25 Hz and an amplitude of 1 to 2.5 cm. The blades’ action on the material produces cavitation waves that effectively grind calcium hydroxide into particles from 100 nm to several microns. Activated lime produced under these conditions is characterised by a high carbonation rate and is used as a catalyst for Portland cement hardening. The advantages of this technology are high productivity and low energy consumption, as the vibrating plant operates in a resonant mode. The disadvantage is the still low yield of calcium hydroxide nanoparticles, which is between 25 and 30%.

Asikin-Mijan et al. [70] prepared lime nanoparticles by firing and subsequent hydration of a product obtained from fresh clams (Meretrix meretrix) from Selangor beach, Malaysia. The clams fired at 800 °C were hydrated with ethylene glycol solution and then treated with diethyl ether (DE) (1 M) and N-Cetyl-N,N,N-trimethylammonium bromide (CTAB) (1 M). Nanoparticles with sizes of 25–42 nm and a specific surface area of 72 m^2^/g were obtained.

Salvadori and Dei [71] investigated the method of preparation of Ca(OH)_2_ nanosized particles performed at a high temperature with use of diols as the reaction media. The method comprised the hydrolysis of calcium chloride solutions in diols (1,2-ethanediol or 1,2-propanediol) by the addition of aqueous sodium hydroxide at temperatures of 150 °C or 175 °C. In addition to the reaction temperature, the influence of other parameters on the results was studied, including the concentration of the reactants and their molar ratio (NaOH concentration 0.17–1.5 mol/dm^3^, CaCl_2_ concentration 0.10–0.75 mol/dm^3^ and NaOH/CaCl_2_ molar ratio 1.2–2.0), as well as the aging time (5 or 40 min). It was observed that the particles obtained had a tendency to agglomerate. Consequently, peptization in an ultrasonic bath with water or 2-propanol as the medium was conducted in the subsequent stage of the experiment. The results indicated that the smallest particle sizes obtained (30–60 nm, see Table 1 for partial results) were achieved with a NaOH/CaCl_2_ molar ratio of 1.4 and an aging time of 5 min. It was observed that the use of 2-propanol as the medium for the ultrasonic bath resulted in enhanced peptization outcomes when compared with water, attributed to the comparatively lower solubility of Ca(OH)_2_ in 2-propanol.

El Bakkari et al. [72] described a method for obtaining calcium hydroxide nanoparticles on cellulose nanofibres. The synthesis was carried out at room temperature using 0.4 M solutions of calcium nitrate tetrahydrate [Ca(NO_3_)_2_·4H_2_O] and natrium hydroxide. After preparation of the lime nanoparticles their carbonation after 90 days of ageing was also studied. The results obtained indicate that the use of cellulose nano-fibres favours the formation of nano-lime particles of a size that is smaller the higher the fibre content in the solution. And the very presence of fibres counteracts the agglomeration of the resulting calcium hydroxide particles and favours the formation of a nano-sized product.

Wang et al. [73] presented a method to obtain nano-lime enhanced with Graphene Quantum Dots (GQDs). The described synthesis method is interesting not only because of the enhancement of the material with GQDs, an idea also presented in their work by Zhu et al. [49], but because of the use of ammoniated CaCl_2_ instead of CaCl_2_ as the calcium hydroxide precursor. Different molar equivalences of ammonia were tested and it was shown that particle size decreased significantly with increasing amounts of ammonia. Particles with an average size of 98.5 nm, 95.7 nm, 85.1 nm and 67.6 nm were obtained when CaCl_2_ (as reference) and CaCl_2_·2NH_3_, CaCl_2_·4NH_3_, and CaCl_2_·8NH_3_ respectively, were used as precursor. In addition, the use of GQDs increased the stability of the obtained suspensions by weakening their tendency to agglomerate.

Chen et al. [74] presented the results of research on the synthesis of calcium hydroxide nanoparticles with the participation of tannic acid. The synthesis was carried out using CaCl_2_ and NaOH as substrates. In the first stage, CaCl_2_ was mixed with a tannic acid solution, which complexed Ca^2+^ ions, and then NaOH was slowly added to the solution. The products of Ca(OH)_2_ precipitation were examined in tannic acid solutions with a mass concentration of 0.01%, 0.03%, 0.05%, 0.1%, 0.3%, and 0.5% and in a reference solution, i.e., in double-distilled water. The nucleation and growth reactions were terminated by adding isopropyl alcohol to the solutions. The tests showed that an increase in the concentration of tannic acid hindered the crystallisation of calcium hydroxide, with clear effects already visible at a concentration of 0.3%. The increase in tannic acid concentration also resulted in calcium hydroxide with a larger specific surface area, and the maximum of this parameter was obtained at an acid concentration of 0.05% (94.09 m^2^/g compared to 0.49 m^2^/g for the reference solution).

### 2.3. Comparison of Synthesis Methods

A concise comparison of synthesis methods is presented in Table 2, summarizing their main principles, particle sizes, advantages, and drawbacks.

Thus, the analysis of literature sources showed that most researchers propose using the ‘bottom-up’ technology to obtain nanolime, where Ca(OH)_2_ nanoparticles are formed as a result of exchange reactions between soluble calcium salts and a precipitant. The resulting precipitate is then cleaned, filtered, and dried. Despite all the positive aspects of this approach, its disadvantages include process complexity, relatively high cost, and limited capability for large-scale production of nanocomposites. At the same time, the ‘top-down’ technology remains promising, especially with the use of various methods of modifying lime quenching processes with surfactants.

As shown in Table 2, bottom-up chemical precipitation remains the most balanced and industrially viable method, offering a combination of high yield, low cost, and scalable processing. However, bio-inspired and mechanically activated (top-down) methods are gaining increasing attention due to their environmental friendliness and alignment with circular economy principles. Future technological development should aim to integrate the advantages of both groups of methods—achieving high productivity and purity while minimizing reagent consumption and environmental impact. Hybrid synthesis approaches, for example, those coupling mild precipitation with mechanical or ultrasonic activation, appear particularly promising for large-scale and sustainable nanolime production.

## 3. Chemical and Physicochemical Properties of Nanolime

The effectiveness of nanolime in construction and conservation largely depends on its chemical and physicochemical properties. This section discusses the structural, morphological, and compositional features of nanolime obtained by different synthesis routes, emphasizing their influence on reactivity and stability.

Only a small number of studies have focused on investigating the properties of nanolime. Michalopoulou et al. [75] studied the morphological characteristics of lime nanoparticles obtained by the ‘bottom-up’ technology. In the case the displacement reaction (CaCl_2_ + NaCl) was used and the greatest attention was paid to the comparison of the morphology of Ca(OH)_2_ particles obtained with use of surfactants: TritonX-100, n-octylamine and amylamine. The reaction medium was distilled water. Using a wide range of investigations (electron microscopy (SEM) FEI Quanta Inspect (FEI Company, Hillsboro, OR, USA), dynamic light scattering (DLS) Malvern Zetasizer Nano Series Instrument (Malvern Panalytical Ltd., Malvern, Worcestershire, UK), Fourier transform, infrared spectroscopy (FTIR) Bruker Tensor 27 spectrometer (Bruker Optik GmbH, Ettlingen, Germany) and X-ray diffraction (XRD) Siemens D-500 diffractometer (Siemens AG, Munich, Germany), it was found that modification of lime with surfactants reduced agglomeration phenomena and changed the morphology of nanoparticles. The addition of Triton X-100 resulted in Ca(OH)_2_ nanoparticles with dimensions between 100–300 nm. The use of Triton X-100 resulted in nanoparticles of smaller size, enhanced width and more angular shape comparing to the products of the synthesis performed without the addition of surfactant. The addition of n-octylamine resulted in plate-like and spherical Ca(OH)_2_ particles, with dimensions between 150–600 nm. Finally, the addition of amylamine resulted in the formation of prismatic, hexagonal and scalenohedral Ca(OH)_2_ nanoparticles, with dimensions between 170 nm and 1 μm. Amylamine resulted in higher diversity in terms of shape and size of the nanoparticle clusters, compared to the uniformity of nanoparticle clusters obtained by the initial bottom-up methodology and the addition of Triton X-100.

**Figure 3:**
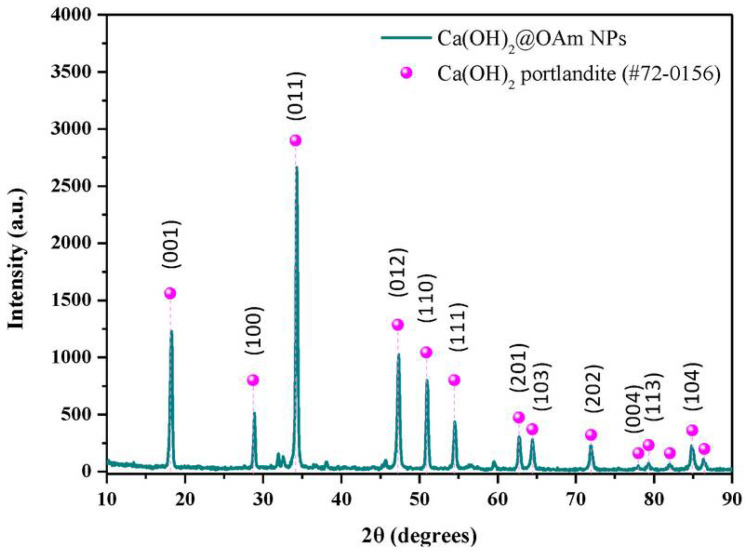
X-ray diffraction pattern of oleylamine-coated calcium hydroxide nanoparticles (Ca(OH)_2_@OAm NPs) obtained via microwave-assisted synthesis [76] (licenced under CC BY 4.0).

**Figure 4:**
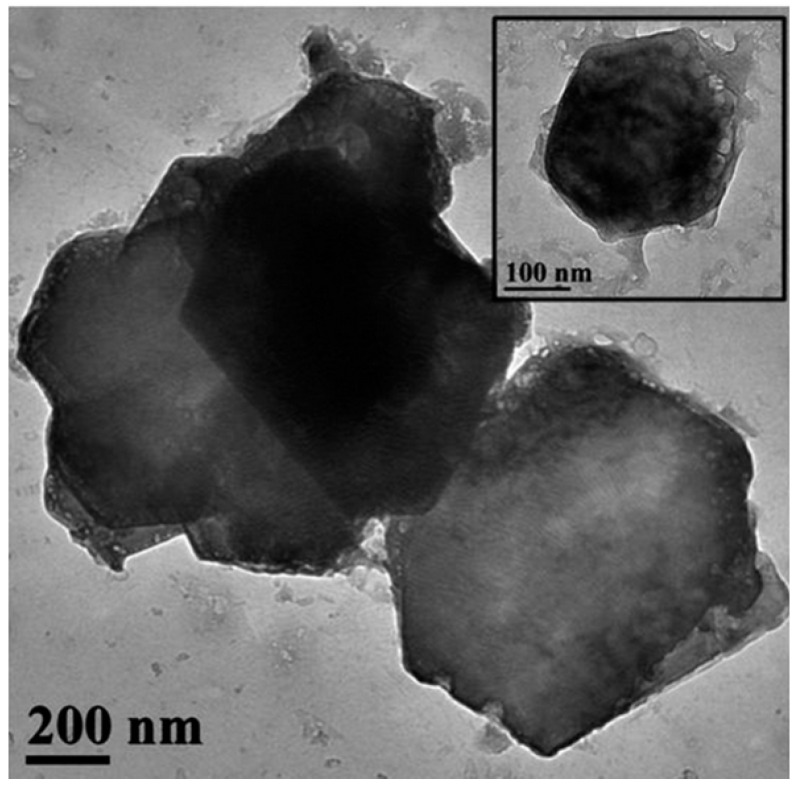
TEM image of hydrophobic calcium hydroxide nanoparticles (Ca(OH)_2_@OAm NPs) coated with oleylamine (scale bar: 200 nm; inset: 100 nm) [76], (licenced under CC BY 4.0).

The hydrophobic calcium hydroxide nanoparticles coated with oleylamine were synthesized using a microwave-assisted process, following the approach described in [76]. The obtained nanoparticles exhibited a well-crystallized hexagonal portlandite structure, as confirmed by XRD (Figure 3), and a uniform plate-like morphology with sizes of approximately 200 nm (Figure 4). The oleylamine coating improved their dispersion stability and reduced agglomeration, which is beneficial for applications requiring controlled carbonation and moisture resistance.

Figure 4 shows TEM images of nanolime particles obtained through various synthesis routes. The micrographs reveal hexagonal and plate-like morphologies typical of portlandite, with particle sizes generally below 100 nm. Differences in particle aggregation and shape reflect the influence of synthesis parameters such as solvent type, pH, and reaction time. The nanoscale size and uniform morphology are key factors contributing to the high reactivity and deep penetration capability of nanolime in porous substrates.

The study by Kilic et al. [13] presented data on the effect of calcium hydroxide on the stability of calcium carbonate particles in aqueous solutions. The electrical conductivity, pH, zeta potential, and average particle size were measured. It was found that the zeta potential of CaCO_3_ particles exceeded +30 mV when placed in Ca(OH)_2_ solution compared to the zeta potential of −10 mV in water. Thus, it was concluded that Ca(OH)_2_ solution is a stabiliser of CaCO_3_ particles. This result shows the superiority of the method for obtaining nano-calcium carbonate by carbonatisation from nano-lime over the method that uses an exchange reaction. During the synthesis of calcium carbonate nano-particles, unreacted nano-lime particles will act as a stabiliser preventing agglomeration of the product obtained.

## 4. Application of Nanolime in Construction Materials

Following the discussion on synthesis methods and material properties, this section examines the practical implementation of nanolime in construction materials. It explores how the specific characteristics of nanolime contribute to enhancing the performance of cementitious composites, asphalt mixtures, autoclaved products, and self-healing systems.

**Figure 5:**
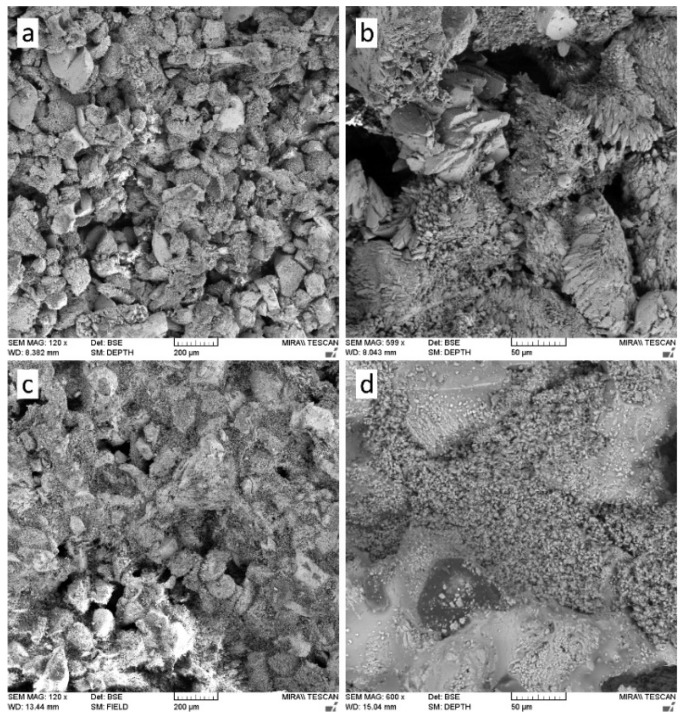
SEM images comparing the microstructure of limestone before (**a**,**b**) and after (**c**,**d**) nanolime treatment, highlighting pore filling and structural consolidation [10].

The application of nanolime in construction materials has attracted increasing attention due to its unique nanoscale properties, which include high dispersibility, large specific surface area, and pronounced chemical reactivity. These characteristics enable nanolime to act as a multifunctional additive, influencing hydration kinetics, microstructural development, durability, and interfacial behaviour. Its action mechanisms can be broadly categorized as: (i) provision of nucleation sites that accelerate hydration and carbonation reactions [2,77]; (ii) filler effects associated with the refinement of pore structures (Figure 5) [3,10,62]; and (iii) supply of reactive Ca^2+^ ions that enhance pozzolanic and carbonate phase formation [6]. As a result, nanolime has been tested in a wide variety of systems including cement and concrete [78,79], lime–pozzolan binders [2,53], autoclaved aerated concrete [80,81], asphalt mixtures [29,31,34,36], and emerging self-healing composites [37,39,82].

In ordinary Portland cement systems, nanolime accelerates hydration reactions by providing abundant nucleation sites for calcium silicate hydrate formation, thereby refining pore structures and reducing capillary porosity [25,26]. When used in combination with supplementary cementitious materials such as fly ash, slag, or calcined clays, nanolime compensates for the lower portlandite availability of low-clinker binders, sustaining pozzolanic activity and improving microstructural densification [25].

Kadhum and Owaid [78] demonstrated that replacing part of the cement with metakaolin enriched with nanolime led to significant mechanical improvements in self-compacting high-performance concrete, with compressive and flexural strength increases of 20–25%. This was attributed to accelerated pozzolanic reactions and denser microstructures. Tudjono et al. [79] investigated the combined effect of nano-fly ash and nanolime on cement mortars, reporting synergistic gains in strength, especially at early ages, due to enhanced pore filling and the formation of additional hydrate phases. These findings highlight nanolime’s potential both as a direct strength enhancer and as an activator in blended cement systems.

In lime-based binders, the incorporation of nanolime significantly enhances reactivity with siliceous and pozzolanic components. The nanoscale dimensions of Ca(OH)_2_ particles increase their dissolution rate and provide a larger surface area for ion exchange, thereby accelerating secondary pozzolanic reactions [2,4,27]. This results in the formation of additional calcium silicate hydrates and calcium aluminate hydrates, which refine pore structures, reduce permeability, and improve overall durability [3,7].

Compared with conventional lime, nanolime penetrates more effectively into porous matrices, ensuring a more homogeneous distribution of reaction products and stronger interfacial bonding between lime and pozzolanic additives [53,83]. These effects are particularly beneficial for sustainable binders with reduced clinker contents, such as lime–metakaolin or lime–calcined clay systems, where the availability of calcium hydroxide is limited [79,84]. In such systems, nanolime not only acts as a reactive source of Ca^2+^ ions but also provides abundant nucleation sites that promote long-term strength development and dimensional stability [79].

Moreover, its ability to carbonate rapidly contributes to further densification of the matrix and additional crack-sealing capacity, enhancing resistance to moisture ingress, freeze–thaw cycles, and chemical attack [2]. Such behavior demonstrates that nanolime can function as both a performance enhancer and a sustainability enabler in next-generation lime–pozzolan binders.

In the production of autoclaved aerated concrete, nanolime has been studied as a reactive lime source that accelerates the crystallization of tobermorite under hydrothermal curing [28,85]. The high surface area and enhanced solubility of nanoscale Ca(OH)_2_ particles provide a rapid supply of Ca^2+^ ions, which facilitates the nucleation and growth of crystalline phases such as tobermorite and xonotlite [86]. This process improves dimensional stability, reduces microcracking caused by differential shrinkage, and results in denser pore walls [87].

The presence of nanolime not only increases compressive strength but also contributes to a more homogeneous distribution of crystalline hydrates, leading to improved long-term performance of AAC [88]. Its ability to act as a controlled lime source is particularly beneficial in low-lime formulations, enabling optimization of the balance between low density and sufficient strength [89]. Recent studies also highlight that nanolime-modified AAC exhibits reduced permeability, further enhancing durability [90]. These findings suggest that nanolime can serve as an effective performance enhancer in AAC systems, where microstructural control is crucial to achieving both lightweight and durable properties.

Applications of nanolime extend beyond cementitious materials to asphalt systems. Nano-Ca(OH)_2_ improves aggregate–bitumen adhesion, mitigates stripping phenomena, and enhances moisture resistance [29,30,31,32]. Experimental results consistently show increased tensile strength ratios, improved retained strength after conditioning, and reductions in moisture-induced damage [34,35]. Additionally, nanolime contributes to improved rutting resistance, fatigue life, and stiffness of asphalt mixtures [91]. Recent studies further indicate that nanolime-modified binders exhibit superior aging resistance and reduced susceptibility to oxidative hardening, leading to more stable long-term performance [92].

Incorporation of nano-Ca(OH)_2_ also improves the viscoelastic properties of bitumen, enhances cohesion within the binder phase, and delays crack propagation under cyclic loading [91]. When used in combination with polymers or nanoclays, nanolime shows synergistic effects, producing asphalt composites with enhanced resistance to permanent deformation and superior durability under harsh environmental conditions [93]. Furthermore, by replacing traditional hydrated lime or liquid anti-stripping agents, nanolime provides an environmentally friendlier solution, reducing material dosage while maintaining or even improving performance [94]. As a result, it has been proposed as a sustainable and multifunctional additive in asphalt pavement technology.

A promising frontier is the incorporation of nanolime into self-healing cementitious systems. Due to its rapid carbonation, nanolime can precipitate CaCO_3_ within microcracks, effectively sealing them and restoring mechanical integrity [2,37,39]. Laboratory studies have further explored embedding nanolime in micro-vascular networks, enabling autonomous and localized delivery to damaged areas [39]. Other research highlights that nanolime can act as a nucleation promoter for calcium carbonate crystallization, accelerating crack closure and contributing to stiffness recovery [7]. These approaches align with the growing demand for durable, low-maintenance infrastructure materials, as they combine the intrinsic benefits of nanolime reactivity with engineered self-healing delivery strategies [95].

The performance of nanolime strongly depends on dispersion media, application protocols, and curing conditions. Alcoholic dispersions (ethanol, propanol, isopropanol) are widely used due to their stability and ability to penetrate porous matrices. Application strategy is also critical: multiple light treatments often yield more homogeneous deposition than single heavy applications [41,96]. Hybrid consolidation methods, such as combining nanolime with ethyl silicate, have been proposed to balance strength enhancement with vapor permeability [42].

Curing conditions, particularly relative humidity and CO_2_ availability, govern carbonation kinetics and polymorph selection. Inadequate curing can lead to incomplete carbonation or superficial crusting, compromising performance [2,96]. Therefore, optimized protocols that control dispersion, dosage, and environmental exposure are essential for achieving reliable results.

Although short-term mechanical and durability benefits of nanolime are well-documented, long-term data remain limited. Critical knowledge gaps exist in understanding nanolime’s performance under aggressive environments such as freeze–thaw cycling, chloride ingress, and sulfate attack [6,62]. Moreover, challenges such as particle agglomeration, heterogeneous distribution, and incomplete carbonation under suboptimal curing continue to hinder broader industrial adoption. Addressing these gaps through systematic testing and field trials will be essential to validate nanolime as a mainstream construction additive.

## 5. Other Applications

### 5.1. Expanding the Use of Nanolime Beyond Construction

Beyond construction, nanolime exhibits a wide range of functionalities that extend into fields such as heritage conservation, environmental protection, energy storage, and medicine. This section summarizes these emerging applications, highlighting how the same fundamental properties of nanolime can be tailored for diverse technological and scientific purposes.

### 5.2. Heritage Conservation

**Figure 6:**
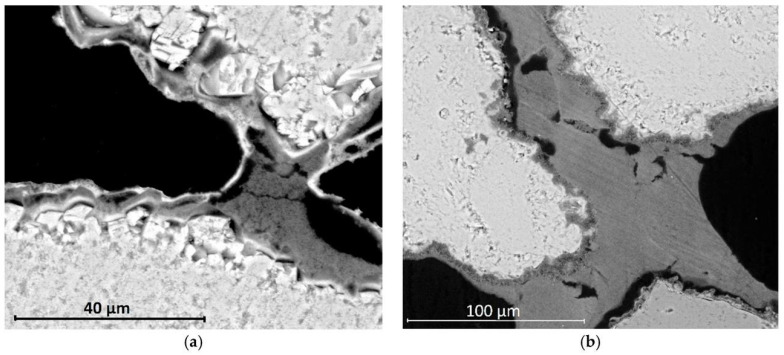
SEM micrographs of limestone showing differences in consolidant application sequence: NL-DAP (**a**) forms nanolime bridges within a detached phosphate layer, whereas DAP-NL (**b**) yields a well-adhered phosphate coating with nanolime bridges linking the grains [109].

The use of nano-sized calcium hydroxide in the restoration of historical monuments is widely documented in the literature [97,98]. In addition to reinforcing stone cladding and structures that have been weakened by the passage of time [99,100,101] and restoring wall paintings [102,103,104], nano-lime has also found other applications that make use of its key and specific characteristics [105,106,107,108] (Figure 6).

Figure 6 illustrates the microstructural differences resulting from the application sequence of nanolime (NL) and diammonium phosphate (DAP) treatments. In the NL-DAP system (Figure 6a), nanolime particles primarily form bridging networks beneath a detached phosphate layer, indicating limited interfacial bonding. Conversely, in the DAP-NL system (Figure 6b), a continuous and well-adhered phosphate coating is observed, with nanolime bridges effectively linking adjacent grains.

Attempts to use nanotechnology in historical restoration have led to the development of products commonly referred to as nanowash. They are used for the conservation of ancient monuments, as well as for strengthening objects containing marble or sandstone with limestone inclusions [110]. The chemical compatibility of nano-lime with the original binder of the above materials has been established and it is assumed that due to its small particle size, it can better penetrate porous material, supplementing the missing binder and thus improving cohesion.

Ambrosi et al. [102] synthesised submicrometric Ca(OH)_2_ crystalline particles by mixing equal volumes of NaOH and CaCl_2_ aqueous solutions with different degrees of supersaturation. In addition to the characterisation of the material obtained by means of SEM, TEM, XRD, among others, the authors also tested the possibility of using it to preserve wall paintings. A successful trial was carried out in the Santa Maria del Fiore Cathedral in Florence on 16th-century wall paintings by Santi di Tito.

Baiza et al. [103] investigated the effect of three consolidants on the colour appearance of *fresco* paint layers affected by lack of cohesion. The materials tested were two types of nanolime: produced in the laboratory according to [102] and commercially available, as well as acrylic resin. Consolidants were applied over two sets of replicas of *buon* and *lime fresco.* The effect on the colour of the replicas was assessed one week and one month after the treatment with technical photography and ultraviolet-induced fluorescence, as well as optical microscopy and colourimetry. The results show no alteration on pigments’ spectral curves and elemental composition. The increase in the CIEL* coordinate and ∆E colour variation was noticed after the treatment with the nanolimes associated with a white haze formation on the paint surfaces.

The use of nanolime for the restoration treatment of fresco wall paintings also carries some risks, such as undesired white veiling deposits. López-Martínez and Otero [104] carried out research into the possibility of eliminating these deposits. For this purpose, fresco wall paintings specimens were prepared in the laboratory and treated with one of the most currently used commercial nanolime products after a certain number of aging cycles. The resulting whitish deposits were removed by five methods, two of which, mechanical cleaning and removal of the excess of product after each application during the treatment, proved the most promising.

Giorgi et al. [111] studied a potential new methodology for the consolidation of wall paintings. For this purpose, dispersions of calcium hydroxide Ca(OH)_2_ in propan-1-ol were used as an alternative to aqueous solutions, which on the one hand contain a small amount of Ca(OH)_2_ due to its low solubility in water, and on the other hand are too unstable to be applied to unprotected painting surfaces. The alcoholic dispersions used were found to be much more stable than suspensions in water and contained three times as much calcium hydroxide. The consolidating power of the applied dispersions was also investigated in this study. The mechanical properties of the specimens were determined using sclerometric resistance and the ‘Scotch Tape test’. The results confirmed that the utilised dispersion provided good consolidation without any glazing on the surfaces. High re-cohesion of the upper paint layers was also observed. In addition to research in the laboratory, the tested dispersion was successfully applied in the restoration of wall paintings by Andrea da Firenze in the Cappellone degli Spagnoli.

Consolidation of mural paintings in hypogea was the subject of the work by Iafrate et al. [112]. The use of organic solvent-based consolidants under such conditions is associated with emissions of volatile organic compounds, which can be particularly harmful in hypogeic environments. Therefore, in the present work, the authors used a new aqueous nanolime dispersion consisting of pure and crystalline Ca(OH)_2_ nanoparticles dispersed in water, produced by an innovative patented procedure. The product was tested in the laboratory and on site, on a medieval mural painting in the Ss. Peter and Paul hypogeum in the UNESCO site of Matera (Italy). The results of the performed pH test showed that carbonisation of the material occurred within 4 h, regardless of whether the process took place in the laboratory or in situ. A new instrument, named ‘spring instrument’, was used to test cohesion, as it shows less risk of damaging the painted layers tested than the traditional scotch tape test. These tests, performed again 4 months after the treatment, showed a lasting re-cohesion effect. The results of colorimetric measurements and tests for potential signs of biodegradation after 4 months were also positive for the tested nanolime consolidant.

Macchia et al. [99] studied the best ways to use calcium hydroxide nanoparticles for the consolidation and restoration of materials in underground environments. Hypogeic environments (underground tombs, caves, catacombs, etc.) are particularly vulnerable due to high humidity, the presence of microorganisms, and the chemical instability of the materials they are built of. Traditional restoration methods can be ineffective or harmful in such conditions. Calcium hydroxide nanoparticles have been shown to be highly effective in strengthening limestone materials.

Otero et al. [113] evaluated the influence of substrate pore structure and nanolime particle size on the effectiveness of nanolime treatments. Two nanolime products were applied by surface brushing of two different types of limestone, which differed, among other things, in pore structure (17.9% of pore volume and 32.88 µm of unimodal pore size versus 14.11% and 34.47 µm). The differences between the formulations used were that the first nanolime product was a dispersion of nanolime in propan-2-ol with a particle size of approximately 100–300 nm, and the second was a 50–50% water/propan-2-ol dispersion of lime particles with sizes of approximately 20–80 nm. The results showed that differences in the pore structure of the treated material may affect the consolidation efficiency depending on the preparation used and its particle size.

Rodriguez-Navarro et al. [60] conducted studies on the effectiveness and possibilities of using alcoholic dispersions of calcium hydroxide nanoparticles for the preservation and restoration of stone monuments subject to chemical and physical degradation. It was found that calcium hydroxide nanoparticles offer a modern solution for the restoration and strengthening of stone surfaces. To protect the surface, dispersions of lime nanoparticles in ethanol were used, which penetrate deeply into porous stone structures. The nanoparticles effectively interacted with calcite to form a stable calcium carbonate, which improved the strength and resistance of the stone to further degradation.

Lanzón et al. [114] investigated the use of dilute suspensions of calcium hydroxide (Ca(OH)_2_) for the restoration and strengthening of historical materials such as stucco, adobe and natural stone. The main focus is on the process of converting Ca(OH)_2_ into nanostructured calcium carbonate (CaCO_3_), which provides increased mechanical strength and long-term stability of the materials. Highly dispersed solutions of calcium hydroxide in low concentration were used to ensure good penetration into the micropores of the materials. Ca(OH)_2_ suspensions penetrated deep pores of the materials, mainly due to the high open porosity of the stone, which facilitated the coating’s penetration, while the low concentration and small particle size of the nanolime suspension further supported this effect. The carbonisation process ensured the formation of uniform CaCO_3_ coatings that were nanostructured and chemically stable. Dilute Ca(OH)_2_ suspensions allow for highly effective strengthening of historical materials due to ease of application and low probability of damage.

Becerra et al. [100] developed and applied a new protocol for evaluating the suitability of selected consolidants for a degraded-stone restoration, which allows for an assessment based on the results of easy-to-perform tests. This protocol was tested on two different consolidants: SiO_2_ nanoparticles and a nanocomposite of Ca(OH)_2_ and ZnO quantum dots, which were applied to carbonated stones. The tests showed that the treatments based on Ca(OH)_2_/ZnO nanoparticles are more recommended for these types of stone, because they do not change the aesthetic and physical properties of the material. This treatment allows the penetration depth to be measured easily and shows the best results in the ultrasound test.

Ricca et al. [101] tested three different consolidating products on stone samples with the same features as the ‘Mendicino stone’ used in the San Domenico church located in the city of Cosenza in southern Italy. The samples were taken from a historical quarry near the city of Cosenza and first treated with one of three consolidants: a commercial suspension of nanosilica, a commercial suspension of nanolime or a laboratory-prepared suspension of nano-calcium hydroxide dispersed in isopropyl alcohol and then mixed with diammonium hydrogen phosphate. The samples were then artificially degraded by salt crystallisation tests and examined, among other things, for the efficacy of consolidating treatments and the resistance of treated stone to salt crystallisation processes. The results indicated greater resistance in the samples treated with nano-calcium-hydroxide mixed with diammonium hydrogen phosphate due to the formation of hydroxyapatite. Almost equally good results were obtained with a commercially available product based on nanolime. However, due to poor compatibility with the reinforced material, significantly worse results were obtained with a commercial product containing nanosilica.

Hoyt et al. [115] used two transparent pore-imitating test systems based on poly(methyl methacrylate) and polydimethylsiloxane to determine the penetration coefficients (PCs) of two commercial nanolimes and their solvents. The PCs were also predicted using material parameters measured and taken from the literature using the Lucas–Washburn equation. The study successfully predicted the infiltration behaviour of alcohols and alcoholic solutions of nanolime, but the results obtained for water-based solutions need improvement. The authors also demonstrated that it is possible to make predictions for the PCs of any desired (also non-transparent) systems, such as a stone for restoration treatments, using PC data obtained from calculations and measurements from transparent systems.

Huesca-Tortosa et al. [116] analysed the effectiveness of various consolidating treatments applied to Pugliese tuff (Gravina Calcarenite), which is a material susceptible to degradation due to its high porosity (approximately 43%), low cohesion and poor mechanical parameters. The study used CaCO_3_ precursors, primarily the bioconsolidant Mixostone M3P and lime water, as well as two nanoparticle-based preparations containing nanosilica and nanolime, respectively. The results showed that the eco-friendly bioconsolidant outperformed the other treatments. In the case of the other three methods, the best results were obtained with the use of nanolime.

López-Arce et al. [117] used non-destructive techniques to evaluate the chemical, morphological, physical and hydric properties of dolostone samples consolidated using slaked lime (Ca(OH)_2_) nanoparticles and then exposed at 33% and 75% relative humidity. The results obtained showed that both at low and high relative humidity levels, an improvement in the properties of the reinforced material was achieved, but high humidity (RH = 75%) is more favourable for dolostone. Under 75% RH, there is a fast transformation of portlandite (Ca(OH)_2_) into vaterite (CaCO_3_), monohydrocalcite (CaCO_3_·H_2_O) and calcite (CaCO_3_). At 33% RH, the main product of consolidation is portlandite, which promotes the recrystallisation of calcite but at the same time causes the dissolution and fracture of dolomite (CaMg(CO_3_)_2_). Hence, it can be concluded that low humidity is beneficial in the consolidation of stones with a high calcite content (e.g., limestone).

Macera et al. [118] presented in their work new aqueous preparations of nanolime for consolidation treatments on biocalcarenite stones used in ancient buildings in the Mediterranean region. Preparations containing pure calcium hydroxide were prepared, as well as preparations enriched with iron (III) ions, in which, in addition to calcium hydroxide, Ca(OH)_2_, tetracalcium ferrite hydrate particles, Ca_4_Fe_2_(OH)_14_·6H_2_O, were also formed. In preparations enriched with iron ions, the weight ratio of Fe to Ca was 1:6, and the particle size of both formed compounds was less than 5 nm. The results of the study showed better compatibility of preparations enriched with iron ions with biocalcarenite stones due to their mineral composition. Regardless of whether suspensions of pure calcium hydroxide or those enriched with iron ions were used, the most favourable results were obtained when the concentration of nanolime in the preparation was 10 g/L, although preparations with concentrations of 5 g/L and 20 g/L were also tested.

Daniele et al. [119] presented in their work new aqueous formulations of nanolime for consolidation treatments of ancient mortars in a hypogeic environment. Preparations containing pure calcium hydroxide were prepared, as well as preparations enriched with iron (III) and magnesium ions. In the latter case, the tests showed that, apart from calcium hydroxide, Ca(OH)2, there was also present magnesium iron hydroxide carbonate hydrate (pyroaurite, Mg_6_Fe_2_(OH)_16_(CO_3_)·4H_2_O), which could have been formed as a result of rapid carbonation of muskoxite, Mg_7_Fe_4_O_13_·10H_2_O during XRD examination. In formulations enriched with iron and magnesium ions, the weight ratio of Mg to Ca and Fe to Ca was 1:4 and 1:10, respectively. The particle size of the pure nanolime formulation was generally in the range of 10–100 nm, and in the enriched one, the particle size was below 50 nm. The results of the study showed the superiority of preparations enriched with iron and magnetite ions over those containing pure calcium hydroxide due to the preservation of the chemical composition and colouring of the original mortar samples. However, both formulations showed similar effectiveness in improving the mechanical properties of treated mortars.

Nanolime is used in the conservation of monuments made mainly of stone materials, but this is not its only area of application, as demonstrated by the publications discussed below. Baglioni et al. [105] presented the specific application of lime nanoparticles in the paper deacidification process and reviewed the literature on this topic. One of them is the work of Giorgi et al. [106] in which nanotechnology was used to prepare dispersions of nano- and microparticles of Ca(OH)_2_ in alcohols, namely propan-1-ol and propan-2-ol. These dispersions were used to deacidify 14th, 17th, 19th, and 20th century acid-yellowed paper samples. It was confirmed that they can be applied to paper using existing procedures, i.e., spraying, brushing, or a combination of both methods. Deacidification using Ca(OH)_2_ alcohol dispersions has many advantages. Thanks to the use of alcohol as a dispersant, with propan-2-ol being particularly beneficial in this role, calcium hydroxide does not dissolve, which could damage the cellulose fibres. At the same time, the dispersion prepared in this way does not hinder the carbonation reaction, which leads to the formation of calcium carbonate, a reservoir of alkalis that protect the paper from degradation.

The same issue is addressed in the work of Bastone et al. [107], in which a nanolime dispersion was prepared by adding propan-2-ol to an aqueous solution of Ca(OH)_2_. The dispersion preparation process was carried out in an inert atmosphere of nitrogen. In order to investigate the effect of temperature and the amount of alcohol on the kinetic stability of the obtained dispersion, the synthesis was performed at different alcohol contents (20, 50, 90 vol.%) and at different temperatures (40, 60 and 76 °C). The influence on particle morphology was also investigated. The results showed that the dispersion prepared at 76 °C with an alcohol content of 90 vol.% exhibited the highest kinetic stability. The smallest particle size was obtained at the same temperature, but with an alcohol content of 20 vol.%. The dispersion with the highest stability was then used to deacidify a document belonging to the *Archivio Storico Diocesano of Palermo* (Italy), demonstrating the high efficiency of nanolime in stabilising pH values and restoring the alkaline reserve in paper.

The procedure for deacidifying historic objects is not limited to books and other paper documents, as demonstrated by Giorgi et al. [108], who described the process of deacidifying wood from the seventeenth-century Swedish warship Vasa. The paper presents the process of deacidifying oak and pine wood samples from the spaceship using a dispersion in propan-2-ol of calcium hydroxide nanoparticles. The traditional washing soda/baking soda method was used as a comparative procedure. The probable cause of the ship’s wood degradation is the presence of sulphuric acid. The dispersion reacts with sulphuric acid to form calcium sulphate, whose particles do not cause additional stress to the wood’s lumens. Excess unreacted calcium hydroxide, which undergoes carbonation, accumulates in the wood, providing a reserve and protection against possible further acid attack. The results obtained showed that the method used produces more lasting effects than the traditional comparative method for both types of wood.

A rather specific application of nano-calcium hydroxide in the conservation of archaeological and palaeontological remains was presented by Natali et al. [120]. They conducted an experiment in which they achieved controlled growth of calcium carbonate crystals, which were a mixture of aragonite and calcite, in order to strengthen demineralised bone remains. The experiment used Ca(OH)_2_ nanoparticles dispersed in propan-2-ol, which were applied to the surface of samples of demineralised bone remains. Crystals on the surface, but also inside the treated deteriorated bones, were formed as a result of the carbonation of calcium hydroxide nanoparticles in the presence of collagen, which served as a template for the slowly growing crystals. As a result of the treatment, the strength of the bones increased by 50–70% compared to the untreated one. Flaking phenomena and powdering were not detectable, leading to a more compact and smooth surface after the applied treatment.

### 5.3. Carbon Dioxide Capture and Environmental Protection

An analysis of studies by many authors shows that nanolime is used as an active component in materials used for environmental purposes. Nuño et al. [121] investigated photocatalytic coatings based on nanostructured calcium hydroxide Ca(OH)_2_ and titanium dioxide (TiO_2_). The mixture of these components effectively decomposes air pollutants such as SO_2_ and NO_2_, and the activity of the coating made from these components increases under the influence of both UV and daylight. In such mixtures, titanium dioxide (TiO_2_) is a catalyst for the carbonisation of nanolime. Experiments have shown that applying these coatings to limestone helps clean surfaces and reduce the impact of harmful gases, which can be used to improve air quality and reduce pollution.

In 1973, Barker [122] showed that the particle size of lime is important for carbon capture. He investigated the reversibility of the reaction CaCO_3_ ⇌ CaO + CO_2_ and came to the conclusion that “the decomposition to the oxide is always 100% but the reactivity of the oxide so formed to carbon dioxide falls off markedly after a rapid initial reaction” [122]. Barker distinguished two phases of the carbon capture reaction by calcium oxide, the first of which, the fast one, takes place on the exposed and highly developed surface of the CaO particles, and the subsequent, slow phase is associated with the diffusion of carbon dioxide through the formed calcium carbonate layer. With increasing reaction cycles, the carbon capture process becomes less and less efficient due to the decreasing size and volume of the pores on the surface of the calcium oxide particles.

Barker concluded that one way to increase the efficiency of carbon capture after many cycles is to use calcium oxide particles with smaller dimensions, in the order of nanometres. He positively verified this hypothesis in his subsequent research [123]. Smaller calcium oxide particles (<40 nm) with a similar specific surface area to the previously used larger particles (approx. 10 μm), but with a less developed surface and less porous structure, showed almost 100% reversibility of the carbon dioxide binding and release reaction. Moreover, they did not show a decrease in performance after more cycles. This is related to the elimination of diffusion as an important mechanism in the carbon dioxide capture process.

Wu et al. in their work [124] studied the properties of nano-CaO/Al_2_O_3_ as a high-temperature CO_2_ sorbent. Its properties, including the ability to adsorb carbon dioxide, were compared to the properties of a sorbent prepared from micro CaO/Al_2_O_3_. The research showed that the nano-sorbent had a higher CO_2_ adsorption rate than its micro variant. In addition, the tests showed that a new phase (Ca_12_Al_14_O_33_) was formed in the tested sorbent, which had a positive effect on its durability.

Lu et al. [12] compared the performance of two kinds of carbon dioxide sorbents. They were made by flame spray pyrolysis (FSP), in which the precursor was Ca-naphthenate, and by calcination (CAL) of three calcium compounds. The FSP-made sorbent consisted of nanostructured CaO and demonstrated its superiority over CAL-made ones, which have grains of bigger size and lower specific surface area. Furthermore, nanostructured FSP-made sorbents showed stable CO_2_ absorption performance even after 60 carbonation and calcination cycles, while the performance of CAL-made sorbents began to decline after 10 cycles, and this decline was steady and uniform.

Santos et al. [125] investigated the properties of a stable synthetic sol–gel CaO sorbent. The resulting material had a coral-like morphology forming branches with a particle diameter of 100–200 nm and a high surface area (45 m^2^/g). Its properties were compared with a sorbent obtained by calcination of commercially available calcium carbonate powder. The tests showed that the sol–gel sorbent exhibited a lower decrease in the efficiency of the carbon dioxide sorption reaction. After 14 calcination/carbonation cycles, it was almost constant (0.58 gCO_2_/gCaO), while the sorbent obtained from calcium carbonate lost 75% of its original capacity after the same number of cycles. After 70 cycles, the sol–gel sorbent maintained a residual sorption capacity of 0.24 gCO_2_/gCaO.

Florin and Harris [126] investigated the carbonation characteristics of CaO derived from nano-sized CaCO_3_ (average size 40 nm) in a multi-cycle performance study of nano-sized CaO sorbents in a continuous CO_2_ capture-and-release process. They proposed a qualitative model of the decay asymptote, which is established through multiple capture-and-release cycles. The authors claim that it is valid for all CaO derived CO_2_ sorbents and represents the establishment of an equilibrium between the pore volume and surface area loss during thermal sintering. The authors also confirmed the existence of the ‘self-reactivation’ phenomenon after extending the carbonation time in the 51st cycle from 20 min to 24 h. In the cycles that immediately followed this cycle, the carbon dioxide absorption capacity increased significantly compared to the previous cycles, but after the 100th cycle, this value decreased to that obtained after 50 cycles.

Liu et al. in their paper [127] investigated an improvement in CaO-based sorbents for cyclic CO_2_ capture. The focus was on increasing the stability of CO_2_ absorption over many cycles, reducing the loss of reactivity due to synthesis and carbonate passivation. Innovative methods such as the addition of stabilisers (e.g., Al and Zr oxides), nanostructured systems and modifications based on porous materials are being considered. These developments improve the kinetics of CO_2_ absorption and reduction under high-temperature conditions, optimising materials for industrial emission capture.

Many articles presented in review papers [128,129,130,131] are devoted to the use of nanolime for soil purification from heavy metals, volatile organic compounds and pesticides, and chemical dyes. Particular attention is paid to materials with high reactivity and surface area, in particular, calcium hydroxide nanoparticles. For this purpose, the processes of adsorption, photocatalysis and filtration are used, depending on the pollutant. The papers also emphasise the functionalisation of nanomaterials to increase their selectivity and stability in complex environments.

For example, Canle et al. [130] reviews the use of nanomaterials to clean the environment of various pollutants, such as heavy metals, organic substances, toxic gases, bacteria, and viruses. Due to their unique physicochemical properties, nanomaterials demonstrate significantly better efficiency compared to traditional methods due to their high reactivity. Various forms of nanoparticles, including metals, metal oxides and hydroxides, carbon nanotubes, graphene, and polymer nanocomposites are discussed. They are used as adsorbents, catalysts, and membranes for water, air, and soil purification. The article describes the use of nanolime for the removal of heavy metals (arsenic, lead, mercury) and organic pollutants (hydrocarbons, pesticides). The effectiveness of nanocomposites based on nanolime for neutralising toxic substances in industrial wastewater and drinking water is noted. Nanolime has been shown to be effective in absorbing toxic gases such as SO_2_, NOx and volatile organic compounds. It can be used in filter systems to reduce air pollution.

A little-known and not very common potential application of nano-sized Ca(OH)_2_ is its use as a photocatalyst. It was tested in this role by Zhang [132]. He synthesised nano-sized calcium hydroxide by a precipitation method. The average size of the particles was about 52 nm. The as-obtained Ca(OH)_2_ samples absorbed light in the visible range and were tested in photocatalytic degradation activities against methylene blue aqueous solution. The results showed that nano-sized Ca(OH)_2_ has great potential for use as a photocatalyst.

Nano-sized calcium hydroxide was also investigated by Narayan et al. [133]. In their study, they used Ca(OH)_2_ synthesised by a precipitation method from the materials derived from sea shells. Prepared samples were immobilised in Alginate beads and they showed excellent photocatalytic degradation activities against methyl red aqueous solution. The catalyst was activated using UV light radiation.

New fields of application for nano-sized calcium hydroxide are constantly being discovered. This is exemplified by the work of Tryfon et al. [134], which presents the results of a study on the effect of calcium hydroxide nanoparticles on plant photosynthesis. In the study, the effects of oleylamine-coated calcium hydroxide nanoparticles on photosystem II (PSII) photochemistry were investigated. The particles were synthesized via a microwave-assisted method and have crystallite size of 25 nm. The study was conducted on tomato plants that were sprayed with Ca(OH)_2_ nanoparticles. The control group consisted of tomato plants sprayed with distilled water. The results showed that synthesised Ca(OH)_2_ nanoparticles could potentially be used as photosynthetic biostimulants to enhance crop yields.

### 5.4. Long-Term Energy Storage

The development of long-term energy storage technologies is an important technical task in the modern development of sustainable energy systems. The promising technology is the use of a high-energy reaction of CaO hydration [16,17]. It is noted that thermochemical heat storage systems are a promising new technology for converting and storing concentrated solar energy. The exothermic lime quenching reaction [CaO + H_2_O] is considered one of the most effective solution and a considerable number of papers have been dedicated to this significant issue [14,15,16,17,18,19,20].

The utilisation of nano-particles of lime has been hypothesised to enhance the efficiency of energy storage; however, the propensity for agglomeration of the particles, particularly in smaller sizes, has been identified as a significant challenge. One of the solutions currently employed to address this issue is calcium hydroxide pelletisation, a countermeasure to the concept of utilising lime nanoparticles. However, this approach does not effectively resolve the problem, as cyclic dehydration and rehydration result in particle disintegration and re-aggregation, leading to an increase in heterogeneity of the bed parameters and a reduction in efficiency [135]. Consequently, alternative solutions are currently being investigated, one such solution being explored by Roßkopf et al. [20]. In this study, the researchers examined a technique for coating bed particles with silicon dioxide (SiO_2_) nanoparticles, marketed under the name Aerosil^®^. The findings were highly encouraging, potentially paving the way for the utilisation of nano-lime, a calcium hydroxide particle with a high specific surface area and reactivity, in long-term energy storage systems. The employment of nano-lime is a promising avenue for exploration, given its potential to enhance process efficiency.

An important element necessary for the implementation of lime and nano-lime in long-term energy storage is research to determine the optimal conditions for the reversible reaction:

Ca(OH)_2_(s) + ΔH_r_ ⇔ CaO(s) + H_2_O(g) (3)

Schaube et al. [14] studied the thermochemical properties of the lime hydration reaction, as well as the physical properties of the resulting substances, such as heat capacity, thermodynamic equilibrium, enthalpy, and reaction kinetics. The high cyclicality of the reaction of conversion of CaO to Ca(OH)_2_ and vice versa at elevated partial pressures of water was experimentally confirmed. It has been experimentally established that the reaction is stable at a partial pressure of H_2_O of 95.6 kPa. At a partial pressure of 1 bar, the equilibrium temperature is 505 °C and the enthalpy of the reaction is 104.4 kJ/mol.

The processes of hydration and water loss in fluidised bed apparatus were investigated by Criado et al. [16] with a focus on its application for concentrated solar power plants. The study included an experimental analysis of a 5.5 kW fluidised bed reactor under conditions simulating large-scale operations. The results were integrated into a 1D model of the reactor to verify the experimental data. The reactor operates at gas surface velocities of up to 0.53 m/s and temperatures of up to 500 °C for the thermal decomposition of Ca(OH)_2_.

Pardo et al. [17] describe in detail an experimental study of the reaction in a fluidised bed reactor. Fine Ca(OH)_2_ with a size of less than 4 µm was used for the experiments. Energy storage with high efficiency was observed up to 50 heating and hydration cycles. The average energy density obtained from the experiments was 60 kWh/m^3^ of the solid mixture. The results demonstrated the possibility of implementing thermochemical heat storage of fine Ca(OH)_2_/CaO in a fluidised bed reactor, with the greatest effect achieved when using Ca(OH)_2_ particles approaching nanoscale.

The same reversible reaction of carbon oxide hydration was studied by Anwar et al. [18]. The results were consistent with the findings of Schaube et al. [8]. At a partial pressure of H_2_O of 1 bar, the expected equilibrium temperature was 505 °C, and the reaction enthalpy was 104.4 kJ/mol. Studies have shown the stability of the system for 100 cycles at partial pressures of H_2_O up to 95.6 kPa.

A review article by Nguyen and Bennici [19] explores the important role of additives in improving the performance and durability of thermochemical energy storage (TCES) materials, especially in limestone-based systems. The use of fine additives, such as Al_2_O_3_ and ZrO_2_, to achieve pellet sintering over several cycles is considered. The importance of selecting auxiliary materials that optimise both stability and reactivity is emphasised.

### 5.5. Medicine

The use of nanoscale drug carriers, such as liposomes, and nanorobots for targeted delivery to affected tissues is being investigated [136]. In oncology nanoparticles are used for drug delivery and photothermal therapy. Their unique properties allow them to effectively heat tumor tissue without damaging healthy tissue [137]. Nanomaterials are also used as contrast agents for tumor imaging [138]. Jandt et al. [139] conducted a comprehensive literature review of the most frequently cited scientific papers in the international peer-reviewed journal Dental Materials over the past five years. The review encompassed a range of dental nanomaterials, including nanocomposites, nanoparticles, antimicrobial nanomaterials such as nanosilica, and biomineralisation systems. The review also examined the distinctive features of dental nanomaterials, highlighting their unique properties and the challenges associated with their fabrication.

Nano-calcium hydroxide is described as an antimicrobial agent, pH regulator, and tissue remineralisation stimulator, especially in dentistry and orthopedics [140,141]. A special emphasis is placed on the use of nanolime in bone tissue regeneration, where its properties contribute to matrix repair and induction of cell growth. Calcium hydroxide is effectively used for antimicrobial purposes in endodontic procedures [142,143].

It is known that Ca(OH)_2_ particles obtained by traditional methods are not subject to size control, have a wide size distribution and polygonal shape. Yang et al. [144] have proposed a template-mediated synthesis and a two-step ion exchange to obtain composite Ca(OH)_2_ particles of uniform size. The developed method makes it possible to obtain particles of the same size, which is important for the predictability of their use in various industries. The nanoparticles obtained in this way are proposed to be used as a drug carrier for bone tissue regeneration.

Nanolime is widely used in bone tissue regeneration due to its high biocompatibility, antimicrobial properties and ability to stimulate remineralisation [145]. Calcium hydroxide nanoparticles ensure the formation of a strong mineralised matrix, which serves as the basis for bone cell growth. Due to their nanoscale structure, the particles are able to penetrate the smallest pores of tissues, ensuring uniform remineralisation. Additionally, nanolime helps to optimise the pH at the regeneration site, reducing the risk of infection. The prospects of its use in dentistry and orthopedics for the treatment of bone defects and injuries are considered, emphasising its effectiveness in comparison with traditional methods [146].

## 6. Discussion

Although numerous studies have demonstrated the effectiveness of nanolime as a consolidant and a component in cementitious and calcareous systems, the available research still presents several inconsistencies and unresolved issues. Reported differences often arise from variations in synthesis parameters, precursor purity, solvent composition, and carbonation control during or after production. For instance, while some authors emphasize that alcoholic nanolime dispersions ensure high reactivity and deep penetration [131] others have reported limited stability and rapid agglomeration under similar conditions [3,41]. These discrepancies suggest that even minor changes in synthesis or storage parameters can significantly influence particle morphology and performance.

Another area of conflicting conclusions concerns the role of particle size and crystallinity. Some studies indicate that smaller, less crystalline nanoparticles exhibit higher carbonation reactivity and better consolidation efficiency [54], whereas others report that moderate crystallinity is required to maintain suspension stability and prevent premature carbonation [3,41]. This inconsistency highlights the need for standardized measurement protocols and reproducible synthesis procedures.

Differences are also evident in the reported performance of nanolime treatments in stone consolidation. Several works confirm an improvement in mechanical strength and cohesion in calcareous substrates [147], while others note surface whitening, pore blocking, or insufficient penetration in low-porosity stones [41]. Such contradictory outcomes underscore the importance of optimizing application techniques, including solvent type, concentration, and environmental conditions during treatment.

Moreover, the interaction of nanolime with other consolidants or additives (e.g., diammonium phosphate, silica nanoparticles, or biopolymers) remains insufficiently understood. While some combinations produce synergistic effects improving bonding and durability [3,77], others have shown phase incompatibility or decreased carbonation efficiency [77,148]. Clarifying these mechanisms through microstructural and chemical analysis is essential for designing more reliable hybrid treatments.

Finally, the long-term durability and environmental performance of nanolime-based systems have not yet been comprehensively addressed. Limited data exist on their resistance to thermal cycling, sulfate or chloride exposure, and irradiation. Similarly, large-scale assessments of cost, embodied energy, and CO_2_ balance are rarely performed, preventing a full evaluation of sustainability benefits.

Despite substantial progress, the field still faces challenges in standardization, reproducibility, and long-term validation. Future studies should aim to reconcile contradictory findings through comparative testing under controlled conditions, supported by advanced characterization and modeling tools. Establishing a unified methodological framework would help move nanolime research from isolated case studies toward consistent, evidence-based application in construction and conservation practice.

## 7. Research Perspectives

Although nanolime has already demonstrated great potential in construction engineering and conservation science, its transition to widespread industrial use requires overcoming scientific, technological, and practical barriers. Several promising research directions can be outlined:

- Scalable and sustainable production [2,70]

Most current synthesis methods remain limited by high cost, energy demand, or sensitivity to carbonation. Future work should focus on low-energy, eco-friendly, and waste-derived routes capable of delivering stable nanolime suspensions at industrial scale. Integration of circular economy principles, e.g., using shells, industrial residues, or CO_2_-derived precursors, could reduce environmental impacts. Combining precipitation with ultrasonic or mechanical activation could enhance reaction kinetics and yield, while continuous-flow reactors may allow process scaling under controlled CO_2_ conditions.

- Control of agglomeration and stability [62,77]

Agglomeration remains a major limitation for reliable performance in cementitious systems. Research is needed on stabilizers, surfactants, and surface functionalization strategies that ensure uniform dispersion without compromising carbonation reactivity. Systematic screening of bio-based dispersants, solvent mixtures, and pH-controlled precipitation conditions could help establish optimized formulations for long-term colloidal stability.

- Durability in harsh environments [9,148]

Long-term studies under freeze–thaw cycling, sulfate and chloride exposure, carbonation depth, and irradiation are still scarce. Accelerated aging tests combined with field trials will be crucial to validate nanolime for durable infrastructure. Coupling these experiments with microstructural monitoring (e.g., XRD, MIP, and µCT) and predictive modeling can provide insight into degradation mechanisms and service-life extension strategies.

- Integration into next-generation binders [27]

With the shift toward low-clinker cements, alkali-activated materials, and hybrid binders, nanolime may act as a reactivity promoter and durability enhancer. Research should clarify its role in balancing hydration, pozzolanic reactions, and microstructural densification. Experimental programs combining calorimetry, isothermal curing, and advanced microscopy could reveal the kinetics and interactions of nanolime within blended or alkali-activated matrices.

- Functional and smart applications [37,39]

Embedding nanolime into self-healing composites, responsive coatings, or multifunctional hybrids (e.g., with photocatalytic oxides or graphene quantum dots) could open new applications in pollution mitigation, energy efficiency, and autonomous repair of infrastructure. Future studies should develop scalable routes for incorporating nanolime into polymeric or inorganic matrices and evaluate their long-term mechanical and functional performance.

- Life-Cycle and techno-economic assessment [5,6]

Comparative studies on cost, embodied energy, and CO_2_ balance versus conventional additives are necessary to support real-world adoption. Establishing standardized protocols for synthesis, application, and performance evaluation will also help bridge the gap between laboratory research and field implementation. Integrating life-cycle assessment with pilot-scale production data could generate reliable benchmarks for environmental and economic feasibility.

- Cross-disciplinary expansion [20,136]

An important research perspective concerns the development of more effective and tailor-made application methods that could overcome the limited performance of nanolime when applied to low-porosity substrates. Recent work by Maucourant and O’Flaherty [43] introduced an innovative nano-electro application technique (nEAT), which enhances the penetration and consolidation efficiency of alcoholic nanolime through electro-assisted processes. Such approaches may open new directions in the conservation of dense limestone and marble materials.

Beyond construction and conservation, nanolime shows promise in environmental remediation, thermochemical energy storage, agriculture, and biomedicine. Expanding research in these areas can further highlight its multifunctional character and create synergies between disciplines.

Future investigations should aim to transform nanolime from a niche consolidant and research material into a mainstream, sustainable component of construction technology and beyond. Coordinated efforts linking chemistry, materials science, civil engineering, and environmental studies will be key to unlocking its full potential.

## 8. Conclusions

Nanolime is a promising material in modern chemistry and technology, widely applied across the construction sector as well as ecology, and other fields, owing to its unique nanoscale properties. Its extremely high dispersibility, large specific surface area, and high reactivity enable faster reactions than traditional lime, making it effective in CO_2_ sequestration, activation of building materials, and water and air purification. Continuous improvements in synthesis technologies, ranging from mechanical activation and chemical precipitation to hydrothermal and hybrid methods, allow tailoring of particle size, porosity, and surface area, thereby expanding its functionality and scope of application.

In construction, nanolime is increasingly used as a modifier of cement and concrete mixtures, enhancing strength, durability, and corrosion resistance, while its self-healing capacity opens new opportunities for environmentally friendly technologies, including the restoration of architectural monuments. Its antibacterial properties and biocompatibility also highlight its potential in dentistry, bone regeneration, and infection treatment. At the same time, challenges such as agglomeration, carbonation control, long-term durability under aggressive conditions, high production costs, and limited performance on low-porosity substrates restrict its large-scale adoption.

Based on the analysis of current research and technological developments, the key conclusions regarding the properties, applications, and future potential of nanolime are as follows:

- It combines heritage applications with emerging uses in construction, ecology, medicine, agriculture, and energy technology.
- Its nanoscale structure enables faster carbonation and improved performance compared to traditional lime.
- Its modern synthesis routes (mechanical activation, precipitation, sol–gel, hydrothermal) allow controlled tailoring of particle size, porosity, and surface area.
- It enhances hydration, strength, durability, corrosion resistance, and self-healing of cementitious materials.
- It is effective in CO_2_ sequestration, pollution mitigation, and as a tool for decarbonisation technologies.
- Its antibacterial, bioactive, and biocompatible properties make it suitable for dentistry and regenerative medicine.
- Challenges remain with agglomeration, stability of dispersions, incomplete carbonation, durability in harsh environments, and production costs.
- Future research should target scalable, eco-friendly production methods and explore cross-disciplinary applications (e.g., energy storage, nanotechnology, smart materials).

In summary, nanolime can be regarded as a multifunctional and strategic material at the intersection of tradition and innovation, with great potential to contribute to sustainable development and global environmental solutions.

## Figures and Tables

**Table 1:** Experimental Conditions for the Hydrolysis of the CaCl_2_/Diol System with Aqueous NaOH Solution and Results (partial, based on [71]).

No.	Solvent	Temp. °C	NaOH mol/dm^3^	CaCl_2_ mol/dm^3^	NaOH/CaCl_2_	Aging Time min	Particle Size nm
1	1,2-ethanediol	150	1.50	0.75	2.0	40	60–150
2	1,2-propanediol	150	1.50	0.75	2.0	40	50–120
3	1,2-ethanediol	150	0.70	0.50	1.4	5	30–60
4	1,2-ethanediol	150	0.70	0.50	1.4	40	40–80
5	1,2-propanediol	150	0.70	0.50	1.4	40	60–90

**Table 2:** Overview of nanolime synthesis methods with their advantages and disadvantages.

Method	Principle	Typical Particle Size	Advantages	Disadvantages	Typical Yield	Approximate Cost	Environmental Friendliness	Industrial Feasibility	Ref.
Precipitation	Ca-salt + NaOH in aqueous/alcoholic media	20–350 nm	Simple, low-cost, tunable particle size, high purity	Agglomeration, limited scalability, CO_2_ protection needed	High (≈70–80%)	Low–moderate	Moderate—requires chemical reagents (NaOH, Ca salts)	High—scalable and well-controlled	[4]
Sol–gel	Chelation and controlled hydrolysis (e.g., citric acid)	55–90 nm	Uniform morphology, mass production potential	Requires annealing, complex processing	Moderate (≈60–70%)	High	Low—due to organic solvents and heating	Moderate	[64]
Hydrothermal/solvothermal	Reaction of Ca salts or metal in solvent at T/P	40–800 nm	High crystallinity, stable dispersions, controlled morphology	Specialized equipment, costly, agglomeration in water	High (>80%)	High	Moderate—high energy input	Low–moderate	[62,71]
Resin-assisted ion exchange	Ca^2+^ exchanged with OH^−^ on resins	<100 nm	Mild conditions, uniform particles	Low yield, limited industrial use	Low (≈30–50%)	High	High—mild and solvent-free	Low	[67,68]
Bio-inspired/waste-derived	Biotemplates (sucrose, shells, etc.)	25–200 nm	Sustainable, low-cost, uses natural resources	Variable quality, reproducibility issues	Moderate (≈60–75%)	Low	Very high—uses natural or waste materials	Moderate	[53,70]
Ball milling	Mechanical grinding of lime suspensions	100–350 nm	Direct use of bulk lime, simple concept	Long processing time, broad size distribution	Moderate (≈50–60%)	Low	High—purely mechanical, no solvents	High	[69]
Vibration-assisted attrition	Cavitation/vibration of lime paste	100 nm—few µm	Energy efficient, high reactivity	Low nanoparticle yield, polydisperse	Low–moderate (≈25–40%)	Low	High	High	[55]

## Data Availability

No new data were created or analyzed in this study. Data sharing is not applicable to this article.
